# The Diseases of the Rectum

**Published:** 1883-02

**Authors:** 


**The Diseases of the Rectum**; Including Fistula, Hemorrhoids, Painful Ulcer, Stricture, Prolapsus, etc., with Diagnosis and Treatment. By WILLIAM ALLINGHAM, M. D., F. R. C. S., Surgeon to S Mark's Hospital for Fistula and other Diseases of the Rectum. Fourth revised and enlarged edition, with illustrations. Philadelphia: P. Blakiston, Son & Co., 1012 Walnut Street. Paper cover, 75 cents; cloth, $1.25.

This is the fifth volume of the octavo series published by Blakiston & Co. Unlike "Wood's Medical Library" series, one may obtain any one of the volumes at the merely nominal price of 75c. If the book could not be obtained at less than the price of the English edition ($3.00), we would still advise all our readers who do not possess it to purchase it. We commend it particularly for its practical worth and as one of the most satisfactory guides in the diagnosis and treatment of diseases of the rectum to be had in the language. Already it has been translated into French, Italian, Spanish and Russian, and this fourth English edition has been carefully revised by the author.

